# Fertility-Sparing Treatment and Assisted Reproductive Technology in Patients with Endometrial Carcinoma and Endometrial Hyperplasia: Pregnancy Outcomes after Embryo Transfer

**DOI:** 10.3390/cancers15072123

**Published:** 2023-04-02

**Authors:** Hilary Friedlander, Jennifer K. Blakemore, David H. McCulloh, M. Elizabeth Fino

**Affiliations:** 1 Division of Reproductive Endocrinology and Infertility, Duke University School of Medicine, 5601 Arringdon Park Drive, Suite 210, Morrisville, NC 27560, USA; 2 Division of Reproductive Endocrinology and Infertility, New York University Langone Prelude Fertility Center, New York, NY 10022, USA

**Keywords:** embryo transfer, fertility-sparing treatment, endometrial hyperplasia, endometrial intraepithelial neoplasia, endometrial carcinoma

## Abstract

**Simple Summary:**

Given that younger women with endometrial carcinoma (EMCA)/endometrial hyperplasia (EH) tend to have better prognoses than older women and fertility-sparing treatment continues to be recognized as acceptable management, the importance of understanding pregnancy outcomes in this cohort is paramount in order to provide better and more informed family planning counseling. The purpose of this retrospective cohort study was to investigate pregnancy outcomes following embryo transfer for patients with a known history of EMCA/EH who underwent fertility-sparing treatment. We found that these patients have significantly poorer live birth rates than expected after embryo transfer, even amongst patients utilizing pre-implantation genetic testing for aneuploidy, suggesting that these sub-optimal rates may be related to the previously diseased endometrial environment or the exposure to high-dose progestins.

**Abstract:**

The goal of fertility-sparing treatment (FST) for patients desiring future fertility with EMCA, and its precursor EH, is to clear the affected tissue and revert to normal endometrial function. Approximately 15% of patients treated with FST will have a live birth without the need for assisted reproductive technology (ART). Despite this low number, little information exists on the pregnancy outcomes of patients who utilize ART. The purpose of this study was to evaluate pregnancy outcomes following embryo transfer in patients with EMCA or EH who elected for FST. This retrospective cohort study at a large urban university-affiliated fertility center included all patients who underwent embryo transfer after fertility-sparing treatment for EMCA or EH between January 2003 and December 2018. Primary outcomes included embryo transfer results and a live birth rate (defined as the number of live births per number of transfers). There were 14 patients, three with EMCA and 11 with EH, who met the criteria for inclusion with a combined total of 40 embryo transfers. An analysis of observed outcomes by sub-group, compared to the expected outcomes at our center (patients without EMCA/EH matched for age, embryo transfer type and number, and utilization of PGT-A) showed that patients with EMCA/EH after FST had a significantly lower live birth rate than expected (Z = −5.04, df = 39, *p* < 0.01). A sub-group analysis of the 14 euploid embryo transfers resulted in a live birth rate of 21.4% compared to an expected rate of 62.8% (Z = −3.32, df = 13, *p* < 0.001). Among patients with EMCA/EH who required assisted reproductive technology, live birth rates were lower than expected following embryo transfer when compared to patients without EMCA/EH at our center. Further evaluation of the impact of the diagnosis, treatment, and repeated cavity instrumentation for FST is necessary to create an individualized and optimized approach for this unique patient population.

## 1. Introduction

Endometrial carcinoma (EMCA) is the most common gynecologic carcinoma in high-income countries [1]. Endometrial hyperplasia (EH) is the precursor to EMCA and has several classification systems. According to the 2014 WHO classification, EH is classified as either simple or complex and with or without atypia. Alternatively, based on the 2000 classification system proposed by a group of gynecologic pathologists, EH is classified as either benign endometrial hyperplasia or endometrial intraepithelial neoplasia (EIN) [2,3,4]. A further classification system, the EIN system, is based on the following histomorphologic parameters: glandular crowding, diameter lesion > 1 mm, and cytologic characteristics in comparison to neighboring endometrium [5]. Irrespective of definition, the overall incidence of any type of hyperplasia is rare in women under age 30 years (6 per 100,000 woman-years); however, the rates increase steadily in each 5-year interval between 30 and 54 years of age [4]. Of all cases of EMCA, 2–5% occur in women before the age of 40, and 71% of these cases are in nulliparous women [3,6]. Concurrently, there has been a trend of increased age at first birth for mothers in the United States, and, therefore, more women may be diagnosed with EMCA or EH prior to the completion of childbearing [7]. In fact, the frequency of EMCA or EH found in infertile women undergoing their first in vitro fertilization (IVF) or intracytoplasmic sperm injection (ICSI) cycle is approximately 3% [8]. 

The gold standard, and only definitive treatment for endometrial cancer, is a hysterectomy, which ends the patient’s chance of bearing children [1]. Therefore, definitive surgical management may not be acceptable for reproductive-aged women desiring future childbearing. Generally, women with EH or early clinical stage EMCA have a good survival prognosis [9,10,11]. Additionally, several studies have shown that younger women have better prognoses when compared to older women [9,10,11]. Therefore, with appropriate counseling and patient selection, fertility-sparing treatment (FST) can and should be considered with the goal of pregnancy or gestation in the future. 

The mainstay of FST is progestin therapy, based on its role in stromal decidualization and endometrial thinning [1]. Historically, oral progestin therapy has been the most commonly used progestin preparation. Today, the levonorgestrel intrauterine device is considered first-line therapy [12]. Progestin therapy has repeatedly been shown to be both safe and effective, and after affected tissue has been eliminated, childbearing may be resumed [13,14,15]. Without treatment, the risk of progression from hyperplasia to carcinoma ranges from 1% to up to 15–75%, depending on the initial histology [16]. The risk of progression with treatment varies by treatment modality [17].

Despite the efficacy of FST, many patients will struggle to conceive naturally. In one meta-analysis of 451 women following FST, 309 women attempted conception spontaneously and without the use of assisted reproductive technology (ART), with only 46 women (14.9%) experiencing a live birth [15]. Furthermore, there remains a paucity of data on the pregnancy outcomes after ART in these patients. Importantly, assisted reproduction after a complete response to treatment is not associated with an increased risk of recurrence [18]. Therefore, we sought to evaluate the pregnancy outcomes for patients with EMCA or EH who underwent embryo transfer after FST to aid in the counseling and treatment options for this unique patient population. 

## 2. Materials and Methods

### 2.1. Design

We conducted a retrospective cohort study of all patients who underwent embryo transfer after FST for EMCA or EH between 1 January 2003 and 31 December 2018 at the New York University (NYU) Langone Fertility Center. The study was performed with NYU IRB approval (#s13-00389). All procedures performed in this study were in accordance with the consent process of the institution and in accordance with relevant guidelines/regulations. In accordance with the institution’s consent process and the study’s retrospective nature, informed consent was waived. 

### 2.2. Subjects

Patients were identified through a query that searched all patient records for the terms “endometrial cancer,” “hyperplasia,” “endometrial intraepithelial neoplasm,” “EMCA,” “cancer,” and “carcinoma.” All patient charts identified through the query were reviewed for inclusion for a complete sampling method. Patients were included if they had: (1) a documented diagnosis of either EMCA or EH, (2) received any duration of FST, and (3) had undergone at least one embryo transfer. We excluded patients who: (1) utilized ART but did not yet return for embryo transfer, (2) utilized ART but elected for intrauterine insemination (IUI), or (3) had an embryo transfer that occurred prior to EMCA/EH diagnosis or prior to FST. 

### 2.3. Variables and Statistical Analysis

All variables were collected from the electronic medical record of included patients. Demographic variables collected included age at diagnosis (years), age at egg retrieval (years), body mass index (BMI), gravidity, parity, endometrial diagnosis, type of fertility-sparing treatment, number of oocytes retrieved, number of total resulting embryos, embryo transfer type (fresh or frozen), use of pre-implantation genetic testing for aneuploidy (PGT-A), embryo transfer date, time to transfer from diagnosis (years), endometrial thickness at the time of transfer (mm), and risk factors for endometrial disease. Any missing data were excluded. 

The primary outcome was live birth rate (defined as the number of live births per number of transfers performed). Secondary outcomes included the number of live births, spontaneous abortions, or negative pregnancy tests. Continuous variables were first assessed for normality using the Kolmogorov–Smirnov test. Unless otherwise specified, descriptive data are presented as a median with an interquartile range. Observed outcomes were compared to expected outcomes at our center based on outcomes from 2016–2018. These expected outcomes, derived from 484 untested autologous frozen embryo transfers (FET) and 2062 tested autologous FETs from patients without EMCA/EH, included age of egg at embryo creation, and type of transfer (fresh or frozen, number of embryos transferred, and with or without PGT-A). A subgroup analysis of euploid embryos comparing observed and expected outcomes were also performed. Statistical analysis included the Wilcoxon Signed-Rank Test. *p* < 0.05 was considered statistically significant. 

### 2.4. Embryo Cryopreservation and Warming

Controlled ovarian hyperstimulation (COH) utilizing a GnRH antagonist protocol with the administration of gonadotropins (recombinant Follicle Stimulating Hormone [FSH], Human Menopausal Gonadotropins [HMG], or both) were prescribed for each patient based on their antral follicle count, age, FSH level, and AMH level (if available) as determined by their physician. Follicular growth and maturation were monitored by transvaginal ultrasound and serum estradiol (E2) level. The GnRH antagonist was added when a lead follicle was identified as 13 mm or greater or the E2 was greater than 1000 pg/mL. Either human chorionic gonadotropin (hCG) alone or hCG with leuprolide acetate was used to trigger final follicular maturation with oocyte aspiration scheduled for 35 h after administration. Oocyte retrieval was performed via ultrasound-guided transvaginal aspiration. Both insemination and ICSI were used for the fertilization of oocytes. ICSI was utilized if indicated by semen parameters, but in our center, PGT-A alone is not an indication for the use of ICSI.

Standard laboratory techniques were employed, and embryos were cultured to the blastocyst stage. If PGT-A was desired, trophectoderm biopsy was performed on day 5 or 6 at the blastocyst stage prior to embryo cryopreservation via vitrification. Biopsy analysis was performed by array comparative genomic hybridization (aCGH) or next-generation sequencing (NGS) based on platform utilization at the time of biopsy. Blastocysts of patients who returned for FET underwent standard embryo warming in our laboratory.

### 2.5. Embryo Transfer

Prior to undergoing embryo transfer, patients underwent a uterine cavity evaluation. Patients who pursued a fresh embryo transfer during an IVF cycle after FST had an embryo transfer on either day 3, day 5, or day 6 after oocyte retrieval. Progesterone suppositories were initiated on post-operative day 1 after retrieval and continued until at least the first pregnancy test. A programmed or hormone-replaced embryo transfer protocol was utilized for patients undergoing FET, all of which were blastocyst embryo transfers. As part of that protocol, patients were administered oral estradiol up-titrated from 2 mg/day to 6 mg/day for at least 10 days or until the endometrium measured ≥7 mm in diameter. Progesterone in oil was initiated, and embryo transfer was planned for the sixth day of progesterone administration. Patients were counseled based on their age, blastocyst formation, embryo quality, and ploidy (if applicable), and a shared decision was made to proceed with fresh, frozen, and multiple embryo transfers. All embryo transfers were direct transfers utilizing ultrasound guidance.

## 3. Results

A total of 14 patients, three (21.4%) with EMCA and 11 (78.6%) with EH were included for analysis, with baseline demographics shown in Table 1. Except for one patient (Patient ID #10), all patients were diagnosed with EMCA/EH and underwent FST prior to their first egg retrieval at our center. The time from diagnosis to first embryo transfer was 0.87 (0.53–2.15) years.

Table 2 shows endometrial diagnoses and individual characteristics by patient. Documented risk factors for EMCA and EH included polycystic ovarian syndrome or PCOS (*n* = 10), BMI ≥ 30 (*n* = 3), and oligo-ovulation (*n* = 2). Ten of the 14 patients were nulliparous, and of the four patients who had previously had a clinical pregnancy, only one patient had a live birth. Fertility-sparing treatments prior to embryo transfer included megestrol acetate (*n* = 7), oral progesterone (*n* = 3), levonorgestrel intrauterine device (*n* = 2), norethindrone (*n* = 2) and polypectomy (*n* = 1). Two patients were treated with more than one FST: norethindrone and megestrol acetate (Patient ID #4) and megestrol acetate and a levonorgestrel intrauterine device (Patient ID #12). One patient (Patient ID #13) ultimately required a hysterectomy.

**Figure 1:**
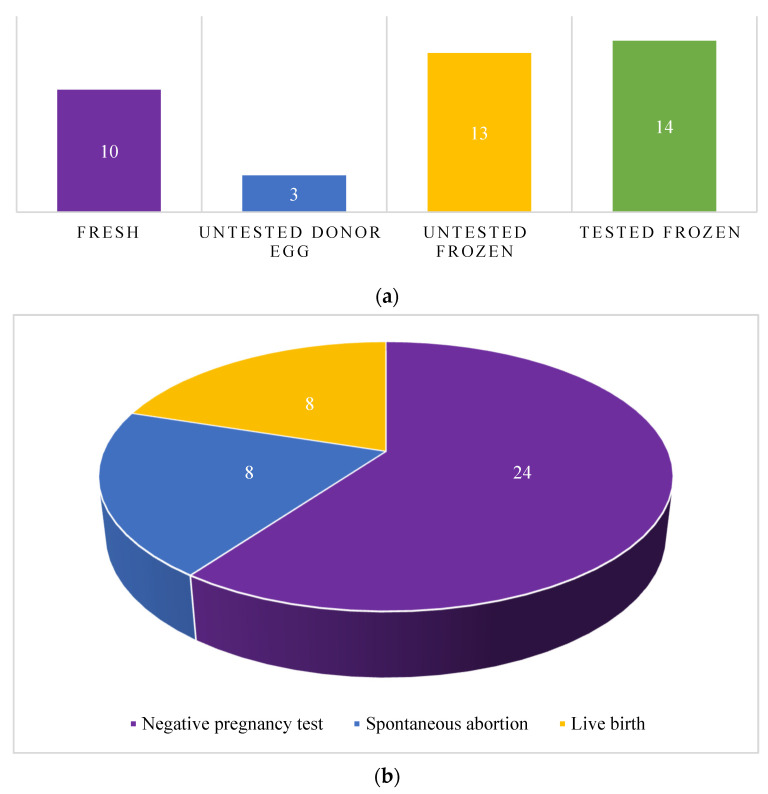

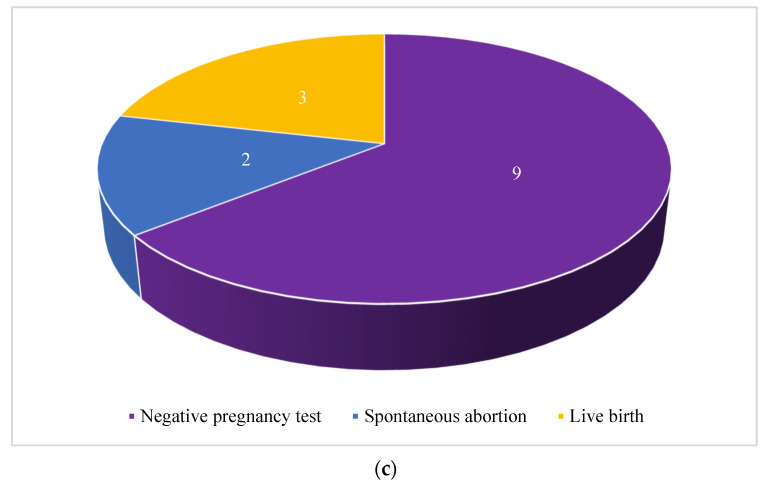
Embryo transfer types and outcomes: (**a**) Total number of embryo transfers by transfer type (**b**) Total number of embryo transfers by pregnancy outcome (**c**) Total number of tested euploid embryo transfers by pregnancy outcome.

The 14 patients in this cohort underwent a total of 40 embryo transfers (Figure 1a). The median number of embryo transfer cycles amongst all patients was 2.5 (2–3), with a median of 2 (1.5–3) and 3 (3–3.5) embryo transfer cycles in the EMCA and EH groups, respectively. Five patients (one with EMCA and four with EH) underwent a total of 10 fresh embryo transfers. Only one of the 10 fresh embryo transfers (Patient ID #7) was a single embryo transfer (SET), and one patient (Patient ID #13) transferred four fresh day 3 embryos in each of her three transfers. Three embryo transfers utilized untested donor eggs, each with a single embryo transferred per cycle and all three from the EH group. Thirteen were frozen autologous untested embryo transfers, with a median of two (1–2) embryos transferred per cycle. SETs accounted for 30.8% of these transfers. All 13 of the frozen autologous untested embryo transfers were performed in EH patients.

Six patients, one with EMCA and five with EH, elected to use PGT-A (Table 3). A total of 14 frozen euploid embryo transfers were performed: seven (50%) single embryo transfers by NGS, four (28.6%) single embryo transfers by aCGH, and three (21.4%) double embryo transfers by aCGH. All three double euploid embryo transfers were in Patient ID #14.

Observed embryo transfer outcomes in our patient cohort included eight live births, eight spontaneous abortions, and 24 negative pregnancy tests. The clinical pregnancy rate per transfer, defined as the number of transfers leading to clinical pregnancy (gestational sac present) over the number of transfers, was 40.0%. The spontaneous abortion rate per transfer, defined as the number of transfers leading to spontaneous abortions over the number of transfers, was 20.0%, resulting in a loss of 50% of the clinical pregnancies (Figure 1b). Observed embryo transfer outcomes for all tested embryos included three live births, two spontaneous abortions, and nine negative pregnancy tests. The clinical pregnancy rate among euploid embryos per transfer was 35.7%, with a spontaneous abortion rate per transfer of 14.3%, resulting in a loss of 40% of the clinical pregnancies (Figure 1c). There were no live births from the 10 embryo transfers performed in the three patients with endometrial carcinoma. 

When comparing the observed live birth rates to the calculated expected live birth rates from matched controls at our center, patients with EMCA/EH after fertility-sparing treatment were found to have a significantly lower live birth rate than expected (Z = −5.04, df = 39, *p* < 0.01). When matching for age and PGT-A platform used based on internal data at our center, a sub-group analysis of the 14 euploid embryo transfers demonstrated a live birth rate of 21.4% which remained significantly lower than the expected live birth rate of 62.8% (Z = −3.32, df = 13, *p* < 0.001). 

## 4. Discussion 

Endometrial carcinoma is the most common malignancy of the female genital tract in the United States [1]. Despite its prevalence, few studies have focused on the pregnancy outcomes after ART in patients who underwent FST [19,20,21,22,23,24,25,26]. We found that patients with EMCA/EH who underwent embryo transfer after FST had significantly lower live birth rates when compared to matched controls at our center. Given that younger women with EMCA/EH tend to have better prognoses than older women and FST remains recognized as acceptable management, the importance of understanding pregnancy outcomes in this cohort is paramount to providing better and more informed family planning counseling. 

Our results supplement prior research. Two studies have shown a low live birth rate in patients with EMCA/EH after ART: (a) a case series examining IVF cycles in patients with stage IA endometrial adenocarcinoma who underwent FST reported a cumulative clinical pregnancy rate of 50.0% and a live birth rate of 27.3% [21] and (b) a retrospective analysis of patients with EH who received FST and IVF had a clinical pregnancy rate of 50.0% and a live birth rate of 38.0% [22]. Interestingly, a case series by Elizur et al., which evaluated IVF outcomes in women conservatively treated for endometrial adenocarcinoma, reported that 75.0% of women conceived and 50.0% delivered healthy offspring [19]. However, a retrospective study by Han et al. of 10 patients who pursued ART in South Korea after remission of disease showed that overall reproductive outcomes after ART might be poor [20]. In this study, four patients underwent IVF and embryo transfer, resulting in one full-term delivery, one pre-viable loss, one pre-term delivery, and one ectopic pregnancy for a live birth rate of 50.0% [20]. 

In patients with both PCOS and EH, Bian et al. investigated the efficacy of levonorgestrel intrauterine devices (LNG IUD) on pregnancy outcomes [27]. Similarly, they found significantly lower clinical pregnancy rates following ART in patients treated with LNG IUD when compared to their control group of patients with PCOS but without EH. In support of the efficacy of progestin therapy for FST, they also found significantly higher clinical pregnancy rates in the LNG IUD group when compared to patients with PCOS and EH not treated with LNG IUD. While interesting, this prospective study only accounts for patients with both PCOS and EH, failing to include all comers with EH and perhaps limiting its generalizability. Besides this aforementioned study, the current literature lacks matched comparisons to patients without treated EMCA/EH, thus missing an opportunity to gain comparative information useful for patient counseling. 

Moreover, to our knowledge, there is a paucity of data specifically evaluating the pregnancy outcomes after euploid embryo transfer in this patient population. Our subgroup analysis of six patients utilizing PGT-A revealed a lower live birth rate than expected based on calculated live birth rates for euploid frozen embryos at our center. Given the importance of the endometrium in implantation, we hypothesize that these sub-optimal pregnancy rates may relate to the previously diseased endometrial environment or the exposure to high-dose progestins. Medroxyprogesterone acetate has been known to reduce the number of glandular cells while also having a negative effect on the decidualization of the stroma [28]. In order to preserve fertility during FST, frequent sampling of the endometrium is required, which could also have an effect on the endometrial environment. This repeated endometrial instrumentation may lead to endometritis, endometrial thinning, or intra-uterine adhesions [28]. For these reasons, patients with EMCA/EH would benefit from collaborative interdisciplinary care between oncology and infertility specialists prior to undergoing FST. 

Our study has several strengths. First, compared to the published literature, a relatively large sample size of patients with EMCA/EH after FST, including multiple FST types. Additionally, we provide concrete clinical pregnancy and live birth rates after euploid embryo transfers, which, to our knowledge, has not yet been published. Therefore, our results forge a deeper understanding of the contribution of endometrial factors in failed implantation. Despite a current emphasis on SET, especially amongst euploid embryo transfers [29], given the large time frame of our study, many patients underwent multiple embryo transfers. The decision to proceed with multiple euploid embryo transfers in Patient ID #14 was an individualized decision based on her family-building plans and oncologic diagnosis. We defined our clinical pregnancy rate as transfers with a gestational sac over the total number of transfers. Given that 21/40 (52.5%) of the transfers were multiple embryo transfers, alternate calculations of clinical pregnancy rates, such as per embryos transferred, may show further differences. 

Limitations of this study include its retrospective and single-center design. Due to the extended time frame included, some demographic data were missing as several of the patients were presented to our center after already completing FST at other institutions in our metropolitan area. During this extended time frame, numerous advances were made in the field of ART. We were unable to control for these advances in our analysis; however, the addition of subgroup analysis comparing only single-thawed euploid embryo transfers does eliminate some of the bias introduced with an extended inclusion time period. Additionally, it is unknown whether patients were treated with metformin as part of their FST regimen, which, when combined with progestin therapy, has recently been associated with higher complete response rates [30,31,32]. Patients who underwent FST followed by spontaneous conception or other forms of ART treatments (e.g., IUI) were excluded. The inclusion of these patients in the future may elucidate further insights into patient decision-making regarding the utilization of ART. Heterogeneity amongst the included patients may limit the generalizability. Future research that separates patients further by histologic diagnosis and necessary treatment (those requiring medical management from those requiring definitive surgical management) would further contribute to our understanding of the impact on fertility. Lastly, future large-scale studies that are prospective, multi-center, and with the inclusion of other ART treatments are necessary in order to not only further characterize outcomes but also to determine better treatment care models for this unique and special patient population. 

## 5. Conclusions

In conclusion, our results demonstrate that women with EMCA and EH represent a special population within the infertility community who require unique care, consideration, and counseling. We have shown that among patients with EMCA/EH who required ART, live birth rates were lower than expected following embryo transfer when compared to patients without EMCA/EH at our center. Even with the transfer of euploid embryos, these poorer outcomes remained. Further evaluation of the impact of the diagnosis, treatment, and repeated cavity instrumentation for FST for EMCA/EH is necessary to create an individualized and optimized approach for this unique patient population.

## Figures and Tables

**Table 1:** Baseline demographics of patients with EMCA/EH after FST presenting for embryo transfer.

	EMCA/EH	EMCA	EH
Patients (*n*)	14	3	11
Median age at diagnosis in years	34 (27–37)	34 (31.5–35.5)	34 (29–38.5)
Median age at retrieval in years	34 (30.75–36.25)	36 (33–38)	34 (31–36)
Median age at first transfer in years	36 (31.25–39.5)	36 (33–38)	36 (31.5–39)
Median BMI	27.88 (20.37–30.9)	35.1 (30.55–36.55)	25.37 (20.12–28.89)
Nulliparity (*n*)	10	3	7
Number of patients using PGT-A (*n*)	6	2	4
Median number of embryo transfer cycles	2.5 (2–3)	2 (1.5–3)	3 (3–3.5)

Note: Data presented as median (interquartile range) unless specified otherwise. FST = fertility-sparing treatment; EMCA = endometrial carcinoma; EH = endometrial hyperplasia; BMI = body mass index; PGT-A = pre-implantation genetic testing for aneuploidy.

**Table 2:** Individual characteristics of patients with EMCA/EH after FST presenting for embryo transfer.

Patient ID	Diagnosis	Age at Diagnosis (years)	Age at Retrieval (years)	Age at First Transfer (years)	BMI	Gravidity/Parity	PGT-A	Risk Factors	Treatment	Total Number of ET Cycles	Pregnancy Outcome		
											LB	SAB	NPT
1	Endometrial hyperplasia	30	31	31	32.06	0/0	No	PCOS	Megestrol acetate	2	1	1	-
2	Endometrial hyperplasia	44	Donor egg	48	19.7	0/0	No	Oligo-ovulation	Progesterone	1	1	-	-
3	Endometrial hyperplasia	40	Donor egg	44	30.9	0/0	No	PCOS, obesity	NA	2	2	-	-
4	Complex hyperplasia without atypia	35	37	37	20	0/0	No	NA	Norethindrone, megestrol acetate	9	-	1	8
5	Complex atypical hyperplasia	28	28	28	29.1	0/0	Yes	Oligo-ovulation	Megestrol acetate	3	-	-	3
6	Complex atypical hyperplasia	34	34	34	20.03	0/0	No	PCOS	Megestrol acetate	4	1	-	3
7	Simple and complex endometrial hyperplasia without atypia confined to polyp	37	38	38	22.85	1/0010	Yes	PCOS	Polypectomy	2	1	1	-
8	Endometrial hyperplasia	27	32	32	20.37	0/0	Yes	PCOS	Norethindrone	1	1	-	-
9	Endometrial hyperplasia with atypia	28	30	30	28.24	4/0040	No	PCOS	Levonorgestrel IUD	3	-	2	1
10	Endometrial hyperplasia	40	34	40	NA	3/2012	No	PCOS	Progesterone	1	-	-	1
11	Endometrial hyperplasia	31	36	36	27.88	2/0020	Yes	PCOS	Progesterone	2	1	-	1
12	Endometrial adenocarcinoma, endometrioid type, FIGO grade I	34	36	36	38	0/0	Yes	PCOS, obesity	Megestrol acetate, levonorgestrel IUD	3	-	-	3
13	Endometrial hyperplasia with a focus on adenocarcinoma	37	40	40	26	0/0	No	NA	Megestrol acetate	3	-	1	2
14	Endometrial adenocarcinoma, endometrioid type, FIGO grade I	29	30	30	35.1	0/0	Yes	PCOS, obesity	Megestrol acetate	4	-	2	2

Note: EMCA = endometrial carcinoma, EH = endometrial hyperplasia, FST = fertility-sparing treatment; ET = embryo transfer; BMI = body mass index, FIGO = The International Federation of Gynecology and Obstetrics, PGT-A = pre-implantation genetic testing for aneuploidy; PCOS = polycystic ovarian syndrome, IUD = intrauterine device, LB = live birth; SAB = spontaneous abortion; NPT = negative pregnancy test; NA = not available.

**Table 3:** Embryo transfer outcomes for euploid embryos.

Patient ID	PGT-A Type	Embryos Transferred (*n*)	Live Birth	Expected Outcome
5	NGS	1	No	0.617
5	NGS	1	No	0.617
5	NGS	1	No	0.617
7	NGS	1	Yes	0.617
8	NGS	1	Yes	0.617
11	NGS	1	No	0.617
11	NGS	1	Yes	0.617
12	aCGH	1	No	0.532
12	aCGH	1	No	0.532
12	aCGH	1	No	0.532
14	aCGH	2	No	0.781
14	aCGH	2	No	0.781
14	aCGH	2	No	0.781
14	aCGH	1	No	0.532

Note: PGT-A = preimplantation genetic testing for aneuploidy; NGS = next generation sequencing; aCGH = array comparative genomic hybridization.

## Data Availability

Data is unavailable due to institution-specific privacy restrictions.
